# Fine Modulation of the Catalytic Properties of *Rhizomucor miehei* Lipase Driven by Different Immobilization Strategies for the Selective Hydrolysis of Fish Oil

**DOI:** 10.3390/molecules25030545

**Published:** 2020-01-27

**Authors:** Maryam Yousefi, Marzia Marciello, Jose Manuel Guisan, Gloria Fernandez-Lorente, Mehdi Mohammadi, Marco Filice

**Affiliations:** 1 Bioprocess Engineering Department, Institute of Industrial and Environmental Biotechnology, National Institute of Genetic Engineering and Biotechnology (NIGEB), Tehran 1497716316, Iran; myousefy@avicenna.ac.ir; 2 Nanobiotechnology Research Center, Avicenna Research Institute, ACECR, Tehran 1936773493, Iran; 3 ‘Nanobiotechnology for Life Sciences’ Group, Department of Chemistry in Pharmaceutical Sciences Dept., Faculty of Pharmacy, Universidad Complutense de Madrid (UCM), Plaza Ramón y Cajal s/n, E-28040 Madrid, Spain; marmarci@ucm.es; 4 Department of Biocatalysis, Institute of Catalysis, CSIC, 28049 Madrid, Spaingflorente@csic.es (G.F.-L.); 5 Departamento de Biotecnología y Microbiología de los Alimentos, Instituto de Alimentación (CIAL), CSIC-UAM, Madrid, Spain

**Keywords:** lipase immobilization, *Rhizomucor miehei* lipase, oriented immobilization, protein chemical modification, fish oil hydrolysis, omega-3 polyunsaturated fatty acids

## Abstract

Functional properties of each enzyme strictly depend on immobilization protocol used for linking enzyme and carrier. Different strategies were applied to prepare the immobilized derivatives of *Rhizomucor miehei* lipase (RML) and chemically aminated RML (NH_2_-RML). Both RML and NH_2_-RML forms were covalently immobilized on glyoxyl sepharose (Gx-RML and Gx-NH_2_-RML), glyoxyl sepharose dithiothreitol (Gx-DTT-RML and Gx-DTT-NH_2_-RML), activated sepharose with cyanogen bromide (CNBr-RML and CNBr-NH_2_-RML) and heterofunctional epoxy support partially modified with iminodiacetic acid (epoxy-IDA-RML and epoxy-IDA-NH_2_-RML). Immobilization varied from 11% up to 88% yields producing specific activities ranging from 0.5 up to 1.9 UI/mg. Great improvement in thermal stability for Gx-DTT-NH_2_-RML and epoxy-IDA-NH_2_-RML derivatives was obtained by retaining 49% and 37% of their initial activities at 70 °C, respectively. The regioselectivity of each derivative was also examined in hydrolysis of fish oil at three different conditions. All the derivatives were selective between cis-5,8,11,14,17-eicosapentaenoic acid (EPA) and cis-4,7,10,13,16,19-docosahexaenoic acid (DHA) in favor of EPA. The highest selectivity (32.9 folds) was observed for epoxy-IDA-NH_2_-RML derivative in the hydrolysis reaction performed at pH 5 and 4 °C. Recyclability study showed good capability of the immobilized biocatalysts to be used repeatedly, retaining 50–91% of their initial activities after five cycles of the reaction.

## 1. Introduction

According to the World Health Organization, a big percentage of all deaths worldwide is related to cardiovascular diseases [1]. Nowadays, many studies indicate that omega-3 polyunsaturated fatty acids (ω-3 PUFAs), derived from fish oil, have a beneficial effect on cardiovascular diseases. Furthermore, the PUFAs, especially eicosapentaenoic (EPA, C20:5 ω-3) and docosahexaenoic acid (DHA, C22:6 ω-3) have nutritional and prophylactic activities, anti-inflammatory effect and exhibit beneficial effects on arthritis, diabetes, and autoimmune diseases [2,3]. Moreover, their important roles in bone growth and brain function and high hypocholesterolemic and hypotriglyceridemic effects of PUFAs have been described [4]. Thus, being the most essential omega-3 polyunsaturated fatty acids, EPA and DHA have received great attention. This is because the key enzymes to produce EPA and DHA are missing in the human body. DHA is an essential compound and has a special function in the natural growth of the brain and retina which is required as an essential nutrient for neuronal functioning and visual acuity in the early stages of life [5]. On the other hand, EPA plays an important role as an anticancer compound and in the prevention of inflammation, arteriosclerosis, autoimmune disorders, and also hypertension and diabetes, in adults [6]. In fact, because of the medicinal benefits of EPA and DHA, various types of fish oil products and supplements from different seafood sources have been developed recently. Most of these commercial products have less saturated fatty acids (SFA) and contain concentrated amounts of EPA and DHA which make them more beneficial than the native oil. Different strategies have been reported for producing PUFA-concentrated products based on chemical or enzymatic methods [7,8].

Within enzymatic approaches, lipase-catalyzed strategies represent a practical methodology due to mild reaction conditions, environmental harmless, and absence of undesirable byproducts [9,10]. Lipases (E.C. 3.1.1.3) have an important role in producing pharmaceuticals, agrochemicals, and natural products due to their high regio, chemo and enantioselectivity [11,12,13,14,15]. Furthermore, high selectivity of lipases permits producing concentrated fish oil products with quite pure samples of EPA or DHA. Nevertheless, the enzyme selectivity in discrimination between EPA and DHA depends on the lipase source and its functional properties. Although there are a few reports of poor selectivity of some lipases, most studies have reported high selectivity of lipases in releasing EPA and DHA in favor of EPA [16,17]. We have also reported the same selectivity in our previous studies of using *Rhizomucor miehei* lipase in selective hydrolysis of fish oil [18,19].

The use of free enzymes in catalytic reactions is limited as they are usually unstable at reaction conditions, and their functional properties can be affected by several parameters (organic solvents, pH, and temperature). Immobilized form of enzymes, on the other hand, encountered great applicability in a broad range of reactions [20]. Up to now, several methods such as adsorption, physical entrapment, adsorption followed by cross-linking, or covalent attachment have been reported for immobilization of lipases [10,21,22]. Enzyme immobilization can impact on catalyst stability and activity also modulating their selectivity in catalytic reactions [23]. However, the functional properties of the final biocatalyst will strictly depend on carrier and immobilization protocol applied for linking enzyme and carrier.

With the aim of finding a proper biocatalyst with improved catalytic properties in order to promote effective production of EPA and DHA from fish oil, we here report the results achieved by immobilizing RML on different sepharose-based supports through different immobilization strategies. For each one, we investigated the effect of immobilization on thermal and co-solvent stability of RML. After that, selectivity of the immobilized preparations in the hydrolysis of EPA and DHA from fish oil was finally investigated in a biphasic system. Finally, the recyclability of immobilized derivatives was also evaluated.

## 2. Result and discussion

### 2.1. Immobilization of RML and Aminated RML on Different Supports

**Figure 1:**
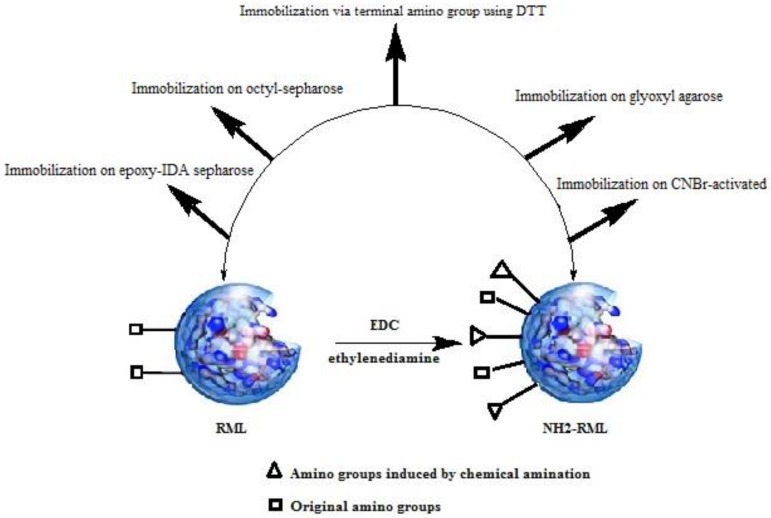
Different protocols for immobilization of *Rhizomucor miehei* lipase (RML) and aminated RML (NH_2_-RML).

RML and chemically aminated RML (NH_2_-RML) were immobilized on different carriers previously activated with different functional groups (Figure 1).

Immobilization of RML on aldehyde functionalized-agarose (Gx-RML) retrieved 47% of immobilization yield after 20 h of incubation at pH 10, 4 °C with 0.6 UI/mg specific activity. The long-time incubation of RML at relatively harsh conditions (pH 10) lowered the specific activity of the final derivative. This is probably related to the low amount of Lys groups in the enzyme surface that limits the enzyme-support multipoint attachment. As previously reported, chemical amination of lipases, after reversible immobilization on octyl-agarose, introduces new primary amino groups with pK_b_ value lower than enzyme original Lys residues, thus increasing the number of possible bonds between the enzyme and support at milder pH values (pH 8–9 approx.). It has been well documented that chemical amination leads to a four-fold increment in the number of amine groups on the surface of RML [24,25]. As a result, the immobilization yields, as well as the derivative stability, are expected to be increased. Effectively, the immobilization of NH_2_-RML on aldehyde functionalized-agarose showed 80% of immobilization yield, which is 1.7-fold higher than immobilization of native RML on this support (Table 1).

After the desorption of both modified and non-modified RML from octyl-agarose, the corresponding free enzymes were also immobilized on CNBr-activated agarose. Immobilization of RML on this support (88%) was performed shortly after 30 min, retrieving a specific activity of 1.9 UI/mg (Table 1). This immobilization protocol is usually carried out to immobilize the free enzyme on a solid heterogeneous support without promoting a multipoint covalent stabilizing immobilization [26]. The linking of the NH_2_-RML enzyme on this support was also performed at the same condition, resulting in only 54% immobilization yield and the specific activity of 1.6 UI/mg (Table 1). Probably, the lower specific activity of the aminated RML after immobilization (CNBr-NH_2_-RML) can be attributed to the deleterious effect of chemical amination of RML on its activity.

Oriented immobilization of RML and NH_2_-RML on epoxy functionalized support was also carried out (Table 1). In this case, the epoxy groups of the support were partially modified by a ring-opening reaction of epoxide moieties with iminodiacetic acid (IDA) followed by chelating the newly introduced carboxylate groups with Ni^2+^ ions (Ni-IDA) [18,27].

This heterofunctional support bearing IDA-chelated Ni^2+^ groups were then used for covalent immobilization of both RML and NH_2_-RML via a two-step mechanism of immobilization. Firstly, the enzyme is adsorbed on the surface of the support by ionic interaction of the imidazole ring of histidine moieties and Ni^2+^ of IDA groups. After that, the covalent attachment of the adsorbed enzyme is performed by promoting the reaction between nucleophilic groups (mainly amine groups) of RML in the neighboring of adsorption site and the epoxy groups of the support.

The structure of RML obtained from the Protein Data Bank (pdb code 3TGL), shows six residues of histidine at the positions of 42,108, 143, 207, 217, and 257. Initial adsorption of the enzyme via ionic interaction is assumed to be driven and oriented by the histidine residue number 42 (H42), which is the only accessible histidine group on the surface of RML. The immobilization yields were 73% and 43% for modified and native RML, respectively producing specific activities of 0.6 UI/mg for RML and 1.1 UI/mg and NH_2_-RML. The strength of RML-support interaction after oriented immobilization was also examined by incubation of the immobilized preparations in a solution containing 300 mM EDTA at 25 °C for 24 h. At this condition, Ni^2+^ ions are expected to be removed by chelation with EDTA molecules leading to desorption of RML molecules. The Bradford and activity assays showed a negligible amount of the enzyme in desorption solution, confirming very strong covalent attachment of RML and NH_2_-RML onto the support.

Immobilization of RML and aminated RML on glyoxyl agarose in the presence of DTT was also performed. It has been previously reported that enzyme linkage on glyoxyl agarose using DTT is directed via the most reactive amine group on the enzyme surface (e.g., the terminal amino) [28].

This strategy allows the enzyme to be immobilized in a milder condition (pH 8, 25 °C), thus preserving the catalytic performances of the final derivatives. After the initial stability improvement, further incubation of the derivatives at higher pH (pH 9–10) would improve the reactivity of both amino groups of the Lys residues and chemically introduced amine groups that finally promote the multipoint covalent attachment of RML. As reported in Table 1, immobilization of RML on this support resulted in a negligible yield (11%), while in the case of modified NH_2_-RML, immobilization yield was 59%, producing specific activity almost four-fold higher than the activity of native RML.

### 2.2. Thermal Stability of the Immobilized Derivatives

**Figure 2:**
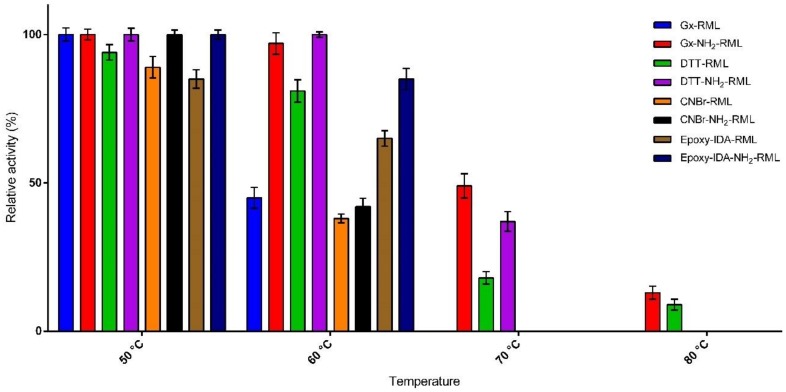
Thermal stability of the immobilized preparations upon incubation at different temperatures for 24 h.

The effects of incubation at different temperatures (50–80 °C) on the activity of immobilized derivatives of RML and NH_2_-RML in phosphate buffer (25 mM) at pH 7.0 were then studied (Figure 2). The preparation obtained by the immobilization of RML on CNBr-activated support was used as reference biocatalyst because of its quite similar properties to the soluble enzyme [29]. At 50 °C most of the derivatives remain completely active during 24 h incubation. Only epoxy-IDA-RML showed lower activity compared to CNBr-RML, showing negative the effect of immobilization of RML via histidine residue.

This observation was in agreement with our previous report on the immobilization of RML on the silica matrix via the same protocol [19]. By increasing the temperature to 60 °C, a further decrease in the activity of the immobilized derivatives was observed. As reported in Figure 2, CNBr-RML loses 62% of its activity. Gx-NH_2_-RML and Gx-DTT-NH_2_-RML were revealed to be the most thermostable preparations showing unaltered specific activities in the same condition. In general, the derivatives obtained from the immobilization of native RML showed lower thermal stabilities compared to the immobilized preparations of NH_2_-RML. These results further confirm the significant effect of multipoint covalent attachment of enzymes on their thermal stabilities. By further increasing the temperature to 70 °C, Gx-NH_2_-RML, Gx-DTT-RML, and Gx-DTT-NH_2_-RML were the only derivatives still active after 24 h of incubation while CNBr-RML lost its whole activity at the same condition. The highest stability at this temperature was shown by Gx-NH_2_-RML with retaining 47% of its initial activity. By incubating these active derivatives at 80 °C, it was observed that Gx-NH_2_-RML and Gx-DTT-NH_2_-RML still retained 13% and 9% of their initial activities, respectively. This great increment in enzyme stability can be attributed to the suitable rigidification of NH_2_-RML on glyoxyl support in these immobilized preparations by multipoint covalent attachment. Glyoxyl agarose has been described as a very suitable support for enzyme-matrix multipoint covalent attachment [30]. In fact, the differences in increased stability can be explained by an increase in the number of support-enzyme bonds that resulted in an intense multipoint attachment.

### 2.3. Stability of RML Derivatives in the Presence of Organic Solvents

**Figure 3:**
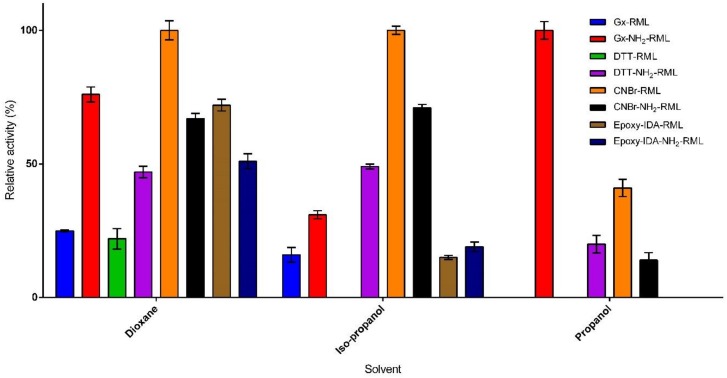
Co-solvent stability of the immobilized preparations in the presence of 20% of 1-propanol, 2-propanol, and dioxane at 25 °C after 24 h incubation.

Organic solvents, particularly those having log P values below 2, can strongly distort the required water-enzyme interaction, thus denaturing the enzyme structure and decreasing its catalytic activity [31]. In order to investigate the effect of immobilization protocol on co-solvent stability, the immobilized derivatives of RML and NH_2_-RML were incubated in the presence of three water-miscible solvents (20% of n-propanol, iso-propanol, and dioxane) for 24 h (Figure 3). The immobilized derivatives showed improved stability compared to the reference derivative (CNBr-RML). In the presence of n-propanol and iso-propanol, CNBr-RML lost its whole activity while it still retained only 22% of its initial activity in the presence of dioxane. Incubation of the immobilized preparations in the presence of dioxane produced different results (Figure 3). Epoxy-IDA-RML, Gx-NH_2_-RML, and Gx-DTT-RML retained 72%, 76%, and 100% of their activities after 24 h of incubation in the presence of dioxane, respectively.

Conversely, Gx-RML, CNBr-NH_2_-RML, and epoxy-IDA-NH_2_-RML lost the major part of their activities in the same condition. Furthermore, a comparison of the obtained results from the co-solvent stability of aminated vs. native RML shows that the strength of enzyme-support linkage is not the only effective factor in the solvent stability of the derivatives. In fact, propanol seems to have the highest deleterious effect on the stability of the immobilized derivatives in which most of the preparations lost their complete activities after 24 h. The only stable derivative was Gx-NH_2_-RML that showed very interesting results while maintaining 100% of its activity (Figure 3). In 2-propanol, the reference derivative was the most unstable preparation, losing entirely its initial activity. On the other hand, the derivatives obtained from the immobilization of RML and NH_2_-RML on glyoxyl by using DTT showed higher stabilities with 100% and 71% residual activities for Gx-DTT-RML and Gx-DTT-NH_2_-RML, respectively.

### 2.4. Fish Oil Hydrolysis

Many researchers have reported enrichment of PUFAs in fish oil by using lipases in free and immobilized forms [32]. It has also been reported that small changes during immobilization strategy can alter the selectivity and activity of the immobilized enzyme. For example, the modulation of lipases selectivity in fish oil hydrolysis has been reported by oriented immobilization [16]. Therefore, the selectivity of the immobilized derivatives of RML was examined in selective hydrolysis of fish oil in order to evaluate the effect of different immobilization strategies on the selectivity of RML. The reaction temperature and pH were the variable parameters assessed in this experiment by applying two temperatures (25 °C and 4 °C) and two pH value (pH 5 and 7). For the quantitation of activity and selectivity, an HPLC–UV analysis was performed. Both parameters were determined by measuring the releasing rate of EPA and DHA during the reaction. All derivatives displayed a significant preference for EPA in comparison to DHA. The observed selectivity has been previously reported for different lipases by several researchers. Table 2 and Table 3 show the results for the hydrolysis reactions in different conditions. The reported activities were calculated by the following equation:

(1) $$\mathrm{Activity}\;=\;\frac{\mathrm{Poly}\;\mathrm{unsaturated}\;\mathrm{fatty}\;\mathrm{acid}\;\mathrm{concentration}\;\left(\mathrm{mmol}\right)}{\mathrm{biocatalyst}\;\left(\mathrm{gr}\right)\times \mathrm{time}\;\left(\mathrm{minute}\right)}$$

The immobilized derivatives of RML and NH_2_-RML presented different results in the reaction based on the type of immobilization procedure (Table 2). For example, a broad range of activities (0.01–1.7) and selectivities (2.7–32.9) were observed by using different procedures and reaction conditions.

As can be seen from Table 2, the highest catalytic efficiency of the derivatives was achieved with the reactions performed at pH 7.0 and 25 °C, presenting the most suitable condition for the application of the immobilized preparations. At this condition, the most active enzyme was NH_2_-RML immobilized on epoxy-IDA with the catalytic efficiency of 1.7 followed by CNBr-RML with a catalytic efficiency of 0.5. The highest EPA/DHA selectivity (18.1) of biocatalysts at pH 7.0 and 25 °C was also obtained for epoxy-IDA-RML. For the other biocatalysts, low to moderate selectivities (ranging between 2.7 and 8.8) were observed. The reduction of pH value from 7 to 5 showed a negative effect on the catalytic efficiency of all the derivatives, while the EPA/DHA selectivities increased if compared to the selectivity values obtained at pH 7, 25 °C. In fact, the highest selectivity (22.1) at this condition was obtained for epoxy-IDA-NH_2_-RML, while its catalytic efficiency was 1.1, which is 65% of catalytic efficiency of this derivative at pH 7, 25 °C. Further investigation was performed on the selected biocatalysts with the highest catalytic efficiency and selectivity to evaluate the effect of temperature on their functional properties (Table 3). The results showed that lowering the temperature caused a remarkable improvement in the selectivity of the selected biocatalysts (CNBr-RM, epoxy-IDA-RML, and epoxy-IDA-NH_2_-RML).

As a general trend, the reduction of the hydrolysis rate of the reaction due to the low temperature allows achieving higher selectivities. As shown in Table 3, oriented immobilization of chemically aminated RML on epoxy-IDA greatly improves its selectivity at pH 5.0 and 4 °C. This improved selectivity accessed the production of almost 97% of pure EPA at the first stages of the reaction.

### 2.5. Recyclability of the Selected Biocatalysts in Fish Oil Hydrolysis

**Figure 4:**
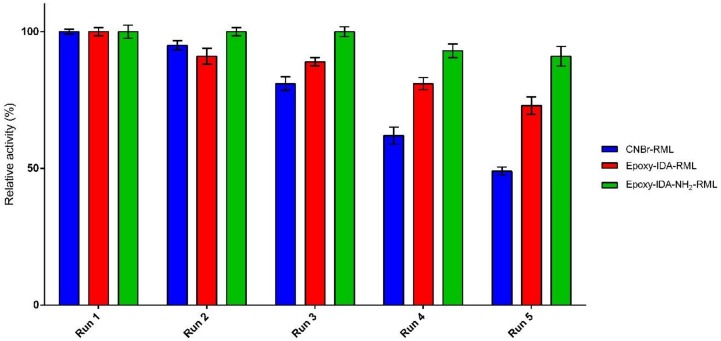
Recyclability of immobilized preparations in fish oil hydrolysis.

Recyclability of an immobilized enzyme is crucial to lowering the process economy in large-scale application of enzymes. The ability of the three selected derivatives for the repeated use in the hydrolysis of fish oil was examined for five cycles. The immobilized lipases were removed by filtration after each run (8 h), washed with cyclohexane, and reused for a new hydrolysis process under the same condition. The enzyme catalytic efficiency in the first cycle of the reaction was set as 100%, and then catalytic efficiency in the subsequent reactions was calculated accordingly. Experiments investigating recyclability indicated good capability of the immobilized biocatalysts to be used repeatedly, retaining 49–91% of their catalytic efficiencies after five cycles of the reaction (Figure 4). For epoxy-IDA-NH_2_-RML in the first three cycles, no loss of catalytic efficiency was appreciated, and after the fifth cycle, the enzyme lost only 9% of its catalytic efficiency.

For epoxy-IDA-RML, after the first two reuses, no relevant loss of catalytic efficiency was observed (−10% approx.), and it decreased up to 70% of retained catalytic efficiency after the fifth use. For CNBr-RML, the initial catalytic efficiency dropped up to the half after five cycles at the same condition. Altogether, the results clearly demonstrated the positive impact of oriented-multipoint covalent immobilization of aminated RML in order to improve the recyclability of biocatalyst in comparison with the results obtained from the of not-aminated RML derivatives.

## 3. Materials and Methods

### 3.1. Materials

Triton^®^ X-100 (TX), 1-Ethyl-3-(dimethylaminopropyl) carbodiimide (EDC), epichlorohydrin, dithiothreitol (DTT), *p*-nitrophenyl butyrate (*p* -NPB), 1,2-ethylenediamine, *cis*-4,7,10,13,16,19-docosahexaenoic acid (DHA), ethylenediaminetetraacetic acid (EDTA) were from Sigma, (Darmstadt, Germany). *cis*-5,8,11,14,17-eicosapentaenoic acid (EPA) was from Cayman chemicals (Ann Arbor, USA). Octyl-sepharose^TM^, CNBr activated sepharose (CNBr) and inert sepharose (Sepharose 6B) were purchased from GE Healthcare (Vienna, Austria). The enzyme from *Rhizomucor miehei* was supplied by Novozymes (Bagsvaerd, Denmark) (Palatase 20,000 U/g lipase, 5.7 mg RML/mL of crude extract), Other reagents and solvents were of analytical or HPLC grade.

### 3.2. Methodology

#### 3.2.1. Purification of RML

Interfacial adsorption strategy was used for the purification of RML from its crude extract [33]. In a solution containing 2 mL of RML (5.7 mg/mL) in 20 mL of sodium phosphate buffer (10 mM, pH 7.0), 1 g of octyl-sepharose was added. Protein concentration in the crude extract and the purified RML were determined using the Bradford’s method [34]. The activity of the supernatant and suspension was spectrophotometrically measured by the method described in Section 3.2.12.

#### 3.2.2. Chemical Amination of Immobilized RML

Solid-phase chemical amination of RML was performed after adsorption of RML on octyl-sepharose [35]. Briefly, 1 g of octyl-RML was incubated in 10 mL of 1 M 1,2-ethylenediamine solution followed by adding of 15.5 mg EDC (10 mM). Then the pH was adjusted to 4.7 and stirred for 90 min. The suspension was finally filtered, and the aminated enzyme was washed with abundant distilled water and stored at 4 °C.

#### 3.2.3. Desorption of the Aminated and Non-Aminated RML from Octyl-Sepharose

For RML purification in its aminated and non-aminated form, desorption of the adsorbed enzyme on octyl-sepharose was performed. For this, 1 g of each derivative was added to 10 mL of sodium phosphate buffer (pH 7.0, 10 mM) containing lauryl sucrose (1%) for non-aminated and Triton X-100 (1%) for aminated RML under gentle shaking for 1 h. Enzyme desorption from octyl-sepharose was monitored by periodically checking the enzymatic activity and concentration in the supernatant and suspension. The purity of each solution was also studied by SDS-PAGE analysis. The pure solutions of RML and NH_2_-RML were used for immobilization on different carriers. In order to prevent diffusion problems, 2.4 mg/g of the pure RML and NH_2_-RML were used in the immobilization process.

#### 3.2.4. Immobilization of RML and NH_2_-RML on Cyanogen Bromide-Activated Support 

The commercial CNBr-activated sepharose was activated prior to use by incubation of 5 g of the support in an acidic aqueous solution (pH 2–3) for one hour [29]. Then, 1 g of the CNBr-activated sepharose was added to 5 mL (0.24 mg/mL) of the purified lipase solution followed by gentle shaking at 4 °C for 1 h. The immobilized derivatives of RML and NH_2_-RML were separated by filtration and washed with 100 mM of NaHCO_3_ at pH 8.0 twice and then re-suspended in 15 mL of 1 M ethanolamine at pH 8.0 for 90 min to block unreacted imido carbonate reactive groups. Finally, the reaction mixture was filtered and washed with abundant distilled water.

#### 3.2.5. Immobilization of RML on Glyoxyl Agarose

For immobilization of the purified RML on glyoxyl agarose, 1 g of the previously prepared matrix was added to 10 mL (0.24 mg/mL) of the enzyme solution [26]. Covalent attachment of RML was performed at pH 10.0 at 4 °C for 24 h under gentle shaking while continuous monitoring of immobilization was performed by the enzymatic assay. Immobilization reaction was ended by adding 10 mg of NaBH_4_ under continuous stirring at room temperature to reduce Schiff’s bases and unreacted aldehydes groups. Subsequently, the final product was filtered and washed with abundant distilled water.

#### 3.2.6. Immobilization of Aminated RML on Glyoxyl Agarose

Immobilization of NH_2_-RML on glyoxyl agarose was performed in two steps. First, 1 g of the carrier was added to the 10 mL of enzyme solution (0.24 mg/mL), and the pH was adjusted to 9.1 by using a saturated solution of Na_2_CO_3_ (10%). After the complete disappearance of the hydrolytic activity of supernatant, the final pH of the solution was adjusted to 10.0, and the suspension was incubated overnight at 4 °C under gentle shaking. Finally, the immobilization reaction was ended by adding 10 mg NaBH_4_ under continuous stirring at room temperature.

#### 3.2.7. Immobilization of Aminated RML via Terminal Amino Group Using DTT

To a solution containing 10 mL of 50 mM DTT in 25 mM sodium phosphate buffer (pH 7.0), 10 mL of NH_2_-RML (0.24 mg/mL) was added [28]. Then 1 g of glyoxyl agarose was added to this solution under gentle shaking at 25 °C followed by increasing the pH first to 9.0 for 3 h and then to pH 10.0 at 4 °C overnight, in order to make multipoint covalent attachment between the enzyme and carrier. Finally, the immobilization process was stopped by adding 10 mg of NaBH_4_ under continuous stirring at room temperature, and then the immobilized enzyme was washed with an excess of distilled water and stored at 4 °C after filtration.

#### 3.2.8. Preparation of Epoxy Support

Epichlorohydrin was used to prepare epoxy activated support, as previously described [36]. Briefly, 5 mL of 6 BCL agarose was washed thoroughly with distilled water and then suspended in 20 mL of 1 M NaOH containing 250 mg of NaBH_4_, 10 mL of acetone, and 5.7 mL of epichlorohydrin. The suspension was stirred for 4 h at 4 °C and finally washed thoroughly with distilled water.

#### 3.2.9. Preparation of the Heterofunctional Support 

One gram of epoxy-functionalized support was incubated in 10 mL of 2 M iminodiacetic acid (IDA) dissolved in 0.1 M sodium bicarbonate buffer pH 11 at 25 °C under gentle stirring [19]. In order to determine the percentage of the modification, samples of the suspension were withdrawn and filtered at predetermined time intervals during the reaction. The resulting support in each time was then washed with deionized water and dried over a sintered glass funnel using vacuum filtration.

#### 3.2.10. Introducing Nickel Cations to the Surface of the Heterofunctional Support

In 10 mL of deionized water containing 0.2 M of NiSO_4,_ 1 g of each partially modified support was added under gently stirring for 2 h. The resulting solid was filtered and washed with an excess of distilled water. In order to quantify the degree of the modified epoxy groups, the nickel cations were released from the support by treatment with 0.5 M EDTA, and then quantification of Ni^2+^ was carried out by atomic absorption spectroscopy.

#### 3.2.11. Orientation and Immobilization of RML and NH_2_-RML on the Heterofunctional Support

One gram of epoxy-IDA was suspended in 10 mL of 25 mM sodium phosphate buffer at pH 7.0 containing a solution of RML or NH_2_-RML (0.24 mg/mL) at 25 °C for 24 h. Periodically, samples of the supernatants were withdrawn and analyzed for protein concentration [16]. After immobilization of the soluble enzymes, the immobilized RML/NH_2_-RML preparations were filtered and washed by distilled water. The immobilized derivative of NH_2_-RML was re-dispersed in 25 mM sodium phosphate buffer at pH 9.0 and incubated at 4 °C for 24 h. In order to remove the metal from the support, the suspension was filtrated and washed several times with a solution containing 0.5 M of EDTA. The final derivative was washed with distilled water and stored at 4 °C.

#### 3.2.12. Enzymatic Activity Assay

The activities of the soluble lipase and its immobilized preparations were analyzed spectrophotometrically by measuring the increment in absorbance at 348 nm (∈ = 5150 M^−1^cm^−1^). The increase in absorbance produced by the release of *p*-nitrophenol (*p*NP) in the hydrolysis of 0.4 mM *p*NPB in 25 mM sodium phosphate buffer at pH 7.0 and 25 °C. To initialize the reaction, 0.05–0.2 mL of the lipase solution (blank or supernatant) or suspension was added to 2.5 mL of substrate solution under magnetic stirring.

#### 3.2.13. Determination of the Amount of Protein Bonded to the Carriers

Protein concentration in supernatant and blank was measured by the Bradford′ s method. The amount of immobilized enzyme was determined as the difference between the remaining enzyme in the supernatant and the protein concentration at the beginning of the immobilization process.

#### 3.2.14. Thermal Inactivation of the Immobilized Preparations

The immobilized preparations RML were incubated in 25 mM sodium phosphate buffer at pH 7.0 at 50 °C, 60 °C, 70 °C, and 80 °C. The suspension of each sample was withdrawn and their activities were measured using the *p*-NPB assay. Each stability reaction was repeated in triplicate and the results have been expressed in a graph as the mean value together with the related SD.

#### 3.2.15. Co-solvent Stability of the Immobilized Preparations

The immobilized preparations were incubated in a total volume of 1 mL solution containing 25 mM sodium phosphate buffer pH 7.0 and 20% of dioxane, n-propanol, and iso-propanol at 25 °C. The suspension of each sample was withdrawn and their activities checked with the enzymatic activity assay as described above. Each stability reaction was repeated in triplicate and the results have been expressed in a graph as the mean value together with the related SD.

#### 3.2.16. Hydrolysis of Fish Oil

A biphasic system was applied for the hydrolysis of fish oil by immobilized derivatives of RML [37]. First, a solution containing 500 µL of fish oil, 5 mL of phosphate buffer (25 mM) pH 5.0 and 7.0 and 5 mL of cyclohexane pre-incubated at 25 °C for 15 min with vigorous stirring. The hydrolysis reaction was then started by adding different immobilized preparations. The concentration of free fatty acids during the reaction progress was determined by taking 100 µL of organic phase at selected time intervals followed by the addition of 200 µL of 2-propanol. Afterward, the selectivity and catalytic efficiency of each derivative were evaluated by using the reverse-phase HPLC (Knauer with a UV detector) on a Grace C_4_ (25 × 0.46 cm). The mobile phase was 55% of acetonitrile/45% of 10 mM ammonium phosphate (*v*/*v*) at pH 3.0 at a flow rate of 0.4 mL/min and 210 nm in the UV detector. The retention times for the unsaturated fatty acids were 32 and 41 min for EPA and DHA, respectively. Each hydrolysis reaction was repeated twice. The results detailed in Table 2 have been expressed as the mean of each experiment set being the standard deviation value lower than 5% in all cases.

#### 3.2.17. Recyclability of Immobilized Derivatives

Recyclability of the selected immobilized preparations was studied by determining the catalytic efficiency of biocatalysts in subsequent reactions relative to that of the first reaction (pH 7.0 and 25 °C). After each cycle (8 h), the biocatalysts were washed with cyclohexane and re-introduced into a fresh reaction medium for another assay run, and this procedure was repeated up to five cycles in the same condition.

## 4. Conclusions

The lipase from *Rhizomucor miehei* is one of the most used enzymes, and it has found many applications in industry. Thus, immobilization of this enzyme can be an interesting way to use it as industrial biocatalyst and finely modulate its catalytic properties upon request. In this paper, we have reported the immobilization of RML and the chemically aminated RML on different activated supports as well as the study of the effect of immobilization protocol on thermal/co-solvent stability of the enzyme. Within the immobilized derivatives, there are some significant differences that depend on the immobilization procedure (e.g., chemical amination of enzyme) and the type of support used. It means that different immobilization protocols permitted us to produce a derivative library showing very different properties.

In more detail, immobilization on glyoxyl-agarose decreased the activity of the native enzyme due to chemical reduction of the imine bond by using NaBH_4_ and also high basicity of the medium (pH 10), which is a condition required to immobilize the enzyme via covalent attachment. Our results showed that the most thermostable preparation is Gx-NH_2_-RML and Gx-DTT-NH_2_-RML, confirming the positive effect of additional amine-mediated multipoint covalent attachment on the enzyme functional properties. These derivatives also showed high stability in iso-propanol and dioxane as co-solvent, whereas in 1-propanol the only stable derivative was Gx-NH_2_-RML. Finally, the derivatives presented quite different behavior in the hydrolysis of fish oil. Nonetheless, all the derivatives discriminate between EPA and DHA in favor of EPA. Regarding the catalytic efficiency of the derivatives in fish oil hydrolysis, the immobilized preparations may be divided into two categories: i) Those showing high catalytic efficiencies with discriminating against PUFAs, and ii) those presenting high selectivities and offering a strong discrimination between EPA and DHA. The derivatives of the first category offered the possibility to use them as biocatalyst to enrich EPA together with DHA in fish oil. On the other hand, the immobilized derivatives with high selectivities can also be used when the concentration of only EPA is needed in the final product. In fact, the epoxy-IDA-NH_2_-RML derivative permitted to achieve the highest catalytic efficiency at pH 7.

## Figures and Tables

**Table 1:** Parameters of different RML preparations.

Enzyme Derivative	Immobilization Yield (%) ^a^	Specific Activity UI/mg Lipase ^b^
Epoxy-IDA-RML	43	0.6
Epoxy-IDA-NH_2_-RML	73	1.1
CNBr-RML	88	1.9
CNBr- NH_2_-RML	54	1.6
Gx-RML	47	0.6
Gx-NH_2_-RML	80	0.5
Gx-DTT-NH_2_-RML	59	0.8
Gx-DTT-RML	11	0.2

Immobilizations were performed as described in the experimental section. ^a^ Yield is defined as the percentage of the soluble enzyme that becomes attached to the support. ^b^ Specific activity is expressed as micromole of substrate hydrolyzed per minute per mg of immobilized protein as described in the experimental section.

**Table 2:** Selective hydrolysis of fish oil by using the immobilized preparations.

Biocatalysts	pH 7, 25 °C		pH 5, 25 °C	
	Catalytic Efficiency ^a^	Selectivity ^b^	Catalytic Efficiency	Selectivity
Gx-RML	0.07	3.6	0.01	6.7
Gx-NH_2_-RML	0.02	2.7	0.01	3.8
CNBr-RML	0.5	3.9	0.16	11.2
CNBr-NH_2_-RML	0.07	8.8	0.04	11.0
DTT-RML	0.01	2.9	0.01	3.7
DTT-NH_2_-RML	0.04	6.6	0.01	2.8
Epoxy-IDA-RML	0.09	18.1	0.08	7.0
Epoxy-IDA-NH_2_-RML	1.7	6.8	1.1	22.1

^a^ Catalytic efficiency is expressed as micromoles of PUFA (EPA and DHA) released per minute and x g of biocatalyst. ^b^ Selectivity is expressed as the ratio between released EPA and released DHA.

**Table 3:** Fish oil hydrolysis by using the selected biocatalysts at pH 5, 4 °C.

Biocatalysts	Catalytic Efficiency	Selectivity
CNBr-RML	0.12	10.6
Epoxy-IDA-RML	0.04	22.1
Epoxy-IDA-NH_2_-RML	0.09	32.9
